# Systemic Inflammation and Astrocyte Reactivity in the Neuropsychiatric Sequelae of COVID-19: Focus on Autism Spectrum Disorders

**DOI:** 10.3389/fncel.2021.748136

**Published:** 2021-11-29

**Authors:** Marta Valenza, Luca Steardo, Luca Steardo, Alexei Verkhratsky, Caterina Scuderi

**Affiliations:** ^1^Department of Physiology and Pharmacology “Vittorio Erspamer”, SAPIENZA University of Rome, Rome, Italy; ^2^Psychiatric Unit, Department of Health Sciences, University Magna Graecia of Catanzaro, Catanzaro, Italy; ^3^Università Telematica Giustino Fortunato, Benevento, Italy; ^4^Faculty of Biology, Medicine and Health, The University of Manchester, Manchester, United Kingdom; ^5^Achucarro Center for Neuroscience, IKERBASQUE, Bilbao, Spain; ^6^Department of Stem Cell Biology, State Research Institute Centre for Innovative Medicine, Vilnius, Lithuania

**Keywords:** astrocytes, autism spectrum disorders, COVID-19, microglia, neuroinflammation, reactive gliosis, astrogliosis, SARS-CoV-2

## Introduction

### The Neurotropism of the SARS-CoV-2

The coronavirus disease (COVID)-19, caused by the severe acute respiratory syndrome coronavirus 2 (SARS-CoV-2), was initially regarded as a specific lung disease. In the course of pandemic evidence for extrapulmonary manifestations has mounted. In particular, neurologic symptoms include anosmia and ageusia, encephalitis, seizures, stroke, confusion and delirium (Desforges et al., 2019; Asadi-Pooya and Simani, 2020; Vaira et al., 2020; Deng et al., 2021; Hugon et al., 2021). Neurological and psychiatric also accompany long-lasting complications of the disease, occurring in patients during the first 6 months after viral infection, while the risk for such sequelae seems to be greatest in case of severe COVID-19 (Fernandez-de-Las-Penas, 2021; Taquet et al., 2021). It has been proposed that the human immune response induced by SARS-CoV-2 develops in two phases. The constitutive adaptive immune response is mobilised at the beginning of the disease confronting actively replicating virus (Shi et al., 2020). A second phase, that occurs in severe cases of COVID-19, is defined as severe acute respiratory distress syndrome (ARDS), characterised by the hyperactivation of the immune system, commonly referred to as “cytokine storm,” with a massive systemic release of proinflammatory mediators, cytokines, and chemokines (Polidoro et al., 2020). This hyperactive immune response and the subsequent cytokine load are now considered among major pathophysiological hallmarks in COVID-19 patients (Abdin et al., 2020). Their impact upon organs, brain including, contributes to the multi-system pathology observed in patients (Gerges Harb et al., 2020; Moore and June, 2020).

Similarly to other members of the group 2 of the β-coronavirus family, SARS-CoV-2 can enter and infest the central nervous system (CNS) (Lau et al., 2004; Bergmann et al., 2006; Steardo et al., 2020a; Zhou et al., 2020). The most studied and acknowledged route for viral entry is through binding to the angiotensin-converting enzyme 2 (ACE2), expressed in the CNS, mostly by endothelial cells, but also found in neurones and neuroglia (Zeisel et al., 2015; Gowrisankar and Clark, 2016; Nemoto et al., 2020). Consistent with the frequent alterations of smell and taste perception reported in COVID-19, SARS-CoV-2 is thought to invade the olfactory system and spread to the brain stem, possibly compromising the respiratory centres (Giacomelli et al., 2020; Lechien et al., 2020; Spinato et al., 2020; Wolfel et al., 2020). The virus could penetrate also through the median eminence, where endotheliocytes and tanycytes express ACE2, thus reaching the hypothalamus (Satarker and Nampoothiri, 2020), and from there spreading to the entire brain. Another possible route is the infiltration of immune cells carrying the virus into the brain [a “viral reservoir” (Iadecola et al., 2020; Tavcar et al., 2021)]. Vessels, meninges, and the choroid plexus have been proposed to act as entry points for infected monocytes, neutrophils, and T cells (Merad and Martin, 2020). However, conclusive evidence of infection through this route is yet to be provided. Lastly, a leaky or dysfunctional blood-brain barrier (BBB) could facilitate the entry of the virus, as seen for other infections (Cisneros and Ghorpade, 2012). Systemic inflammation damages glia limitans and the BBB, thus the hyperreactive immune response triggered by SARS-CoV-2 could compromise the integrity of the BBB (Valenza et al., 2020). Moreover, comorbidities often associated with severe COVID-19, e.g., CNS hypoxia due to respiratory failure, thrombotic microangiopathy, or pre-existing neurological diseases, could have already altered the BBB permeability facilitating SARS-CoV-2 invasion of the brain (Erickson et al., 2021).

### Astrocytes Response to Viral Infections, Including SARS-CoV-2

Any insult to the CNS, including viruses, triggers glial reactivity (Verkhratsky et al., 2017; Zorec et al., 2019; Escartin et al., 2021) aimed at restoring the lost homeostasis. At the same time, during viral infections, astrocytes and microglia may also become long-term viral reservoirs in the absence of efficient innate immune-mediated clearance. Viruses-induced rise in IL-1β and TNF-α may change astrocyte metabolism, thus impairing neuronal energy support (Gavillet et al., 2008; Soung and Klein, 2018). In human immunodeficiency virus (HIV) infection, reactive astrocytes overproduce cytokines and chemokines able to reduce viral replication (Zhou et al., 2004; Li et al., 2011). Broad hyperplasia of glial cells, with necrosis of neurones, and encephalic oedema have been reported in a SARS-CoV-1 patient (Xu et al., 2005). Several case reports indicate that SARS-CoV-2 affects astrocytes. A rise in the glial fibrillary acidic protein (GFAP), commonly regarded as a marker of astrocyte reactivity, was found in the white matter of a COVID-19 patient with encephalomyelitis-like brain damage (Reichard et al., 2020). Plasma levels of GFAP were elevated in moderate/severe stages of COVID-19 suggesting that astrogliosis is an early CNS response to SARS-CoV-2 infection (Kanberg et al., 2020). In a COVID-19-related acute necrotising encephalopathy, 19 days after the onset of symptoms and even after testing negative twice for COVID-19, the SARS-CoV-2 was detected in the CSF together with extremely high levels of both the neurofilament light-chain protein (NfL), a biomarker predictive of intra-axonal neuronal injury, and GFAP (Virhammar et al., 2020). These clinical data indicate that astrocytes enter a reactive state in COVID-19 patients. Moreover, the damage to the BBB and the strong lymphopenia observed during COVID-19 could promote the persistence of the virus into the brain, thus sustaining neuroinflammation and reactive gliosis. The resulting brain tissue alteration could explain some of the clinical features observed in COVID-19 patients who, despite resolved pneumonia, present cognitive impairments associated with behavioural changes (Sasannejad et al., 2019; Steardo et al., 2020b, 2021; Tremblay et al., 2020; Boldrini et al., 2021).

### COVID-19 During Pregnancy

Pregnant women are considered at high risk to develop severe COVID-19, despite case reports indicate that the disease severity is similar to the general population (Mullins et al., 2020; Rasmussen et al., 2020; Zaigham and Andersson, 2020). Infections with SARS-CoV-2 during pregnancy have been associated with preterm delivery, intrauterine growth retardation, and perinatal deaths (Diriba et al., 2020; Huntley et al., 2020; Woodworth et al., 2020; Bellos et al., 2021). A retrospective study shows that SARS-CoV-2 infection during pregnancy is not associated with an increased risk of spontaneous abortion and spontaneous preterm birth (Yan et al., 2020). Studies reported zero to very low rate of vertical transmission from the mother to the foetus. Some case reports highlighted the presence of both M and G immunoglobulins against SARS-CoV-2 at birth in three neonates whose mothers presented with COVID-19 23 days before delivery (Dong et al., 2020; Zeng et al., 2020). In a cohort of 64 pregnant women who tested positive for SARS-CoV-2, 12 had severe to critical COVID-19, but neither placental infection nor vertical transmission occurred (Edlow et al., 2020). In contrast, one case of SARS-CoV-2 transplacental transmission has been reported, in which both the placental tissue and the amniotic fluid were positive as maternal and neonatal blood samples. Of note, mother's infection occurred at the last weeks of gestation (Vivanti et al., 2020).

### COVID-19 and Neuropsychiatric Sequelae: Focus on Autism Spectrum Disorders

Epidemiologic data correlate maternal infections with several neuropsychiatric disorders, including autism spectrum disorders (ASD) (Minakova and Warner, 2018). Autism and ASD are terms indistinctively used to define a group of heterogeneous neurodevelopmental disorders affecting about 1% of the world's population (Elsabbagh et al., 2012; Ilieva and Lau, 2020). Precise aetiology of ASD is still unknown. Both genetic and environmental factors are thought to contribute, including an increase of inflammatory cytokines, abnormal immune responses, and the presence of autoantibodies (Ormstad et al., 2018; Mazon-Cabrera et al., 2019). Some of these features are in common with those considered risk factors for severe COVID-19. Therefore, some authors have speculated that ASD could be a risk factor for SARS-CoV-2 infection and COVID-19 outcome (Lima et al., 2020; Brown et al., 2021).

Numerous environmental factors are thought to increase the risk for ASD, as neurotoxins, air pollutants, and drugs (Riley and McGee, 2005; Grandjean and Landrigan, 2006; Brown, 2012; Krakowiak et al., 2012; Saxena et al., 2020) as well as perinatal infections (Hornig and Lipkin, 2001). Evidence supporting a link between infection during pregnancy and ASD incidence is increasing (Bilbo et al., 2018). A two-fold increase of ASD has been documented following maternal infection with influenza virus, but not with common infections, such as cystitis or genital herpes (Atladottir et al., 2012; Croen et al., 2019). Maternal diagnosis of viral or bacterial infection, regardless of the timing of the infection during pregnancy, has been associated with approximately a 30% increase in ASD risk for their children (Lee et al., 2015). Thus, some authors suggested that prenatal viral infection could represent the principal non-genetic cause of autism (Ciaranello and Ciaranello, 1995; Depino, 2018). To date, there is no evidence documenting a causal link between COVID-19 and ASD. However, this neurodevelopmental disease could be diagnosed few years after birth, thus upcoming reports could provide data for ASD incidence in SARS-CoV-2 infected mothers.

### Reactive Astrocytes in the Foetal Brain: Possible Link to Autism Spectrum Disorders?

**Figure 1:**
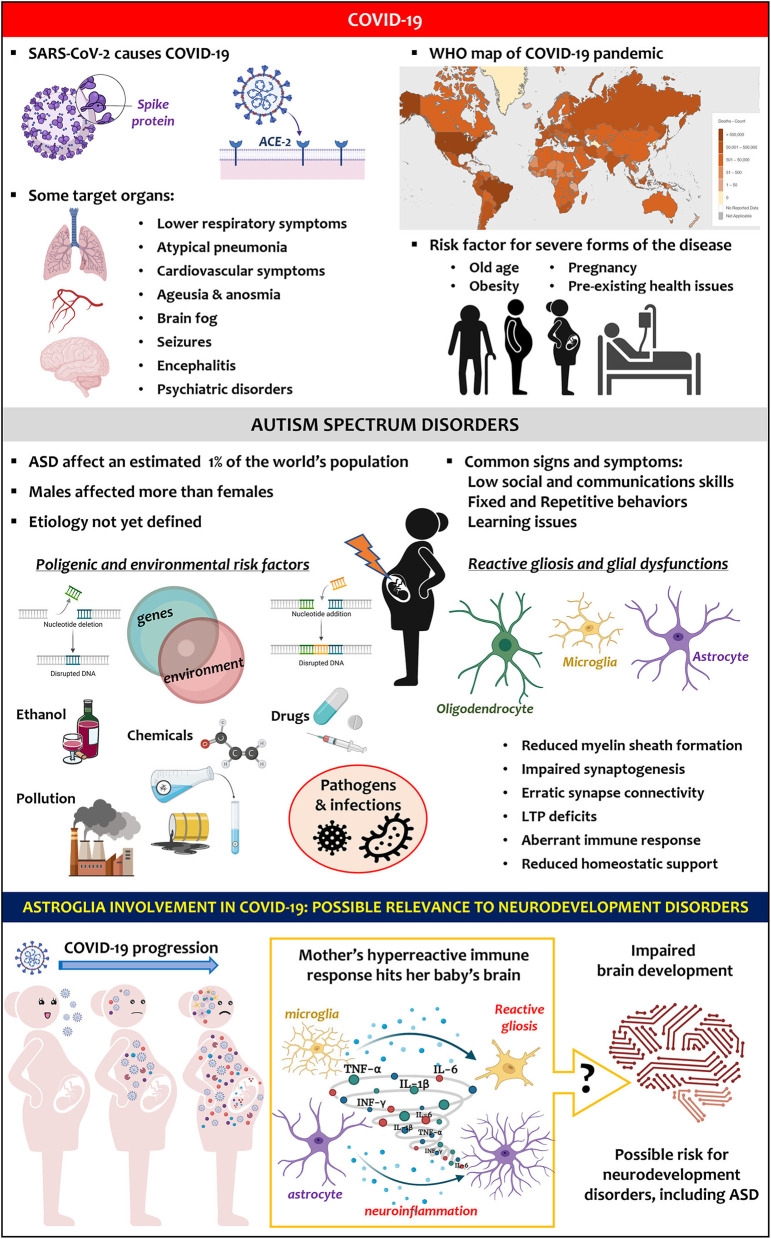
Key facts on SARS-CoV-2 infection and COVID-19 pandemic (upper panel). Key facts on ASD (middle panel). Hypothesis (lower panel): COVID-19-induced hyperreactive immune response in pregnant women could trigger astroglia reactivity in the baby's brain, altering its development and favouring neurodevelopment disorders, including ASD.

Despite the lack of evidence, a link between maternal SARS-CoV-2 infection and ASD can be speculated. It is well known that the perinatal environment markedly affects brain development and function, and, for this reason, some of the cellular and molecular alterations caused by SARS-CoV-2 could hypothetically promote ASD (Figure 1) (Steinman, 2020a; Rasile et al., 2021). Above all, the activation of the maternal immune system with the subsequent exposure of the foetus to high levels of cytokines, chemokines, and other mediators of inflammation through maternal serum, placenta, and amniotic fluid may impact on the brain (Knuesel et al., 2014). Foetal exposure to infections is accompanied by modifications in the expression of proinflammatory mediators, reactive gliosis and altered expression of genes involved in brain development, all previously linked with ASD (Pardo and Eberhart, 2007; Li et al., 2009; Zeidan-Chulia et al., 2014; Liao et al., 2020). Among several cytokines, IL-6 has attracted much attention mainly because it is elevated in cases of complicated forms of COVID-19 and correlates with adverse clinical outcomes (Chen et al., 2020; Zhu et al., 2020). Incidentally, IL-6 plays a key role also in ASD. Data correlated the *in utero* exposure to IL-6 and ASD-related features (Smith et al., 2007). Increased IL-6 levels were detected in the brains of ASD patients compared with controls subjects (Li et al., 2009). An increase in IL-6 placental levels was found to negatively correlate with insulin-like growth factor 1 (IGF-1) (Patterson, 2009). This is relevant to ASD since autistic children below four years old show lower concentrations of IGF-1 than age-matched controls (Riikonen et al., 2006). IGF-1 participates in several physiologically relevant neuroprotective mechanisms and exerts significant effects on foetal and perinatal brain growth, including neurogenesis and synaptogenesis (Steinman, 2020b). The hyperactivation of systemic immune response and specifically the increase in circulating IL-6 in a mother infected by SARS-CoV-2 could expose the foetus to an aberrant inflammatory environment, which is deleterious for the developing brain. As we mentioned before, SARS-CoV-2 triggers astrogliosis and microgliosis fostering remodelling of brain circuits through the synthesis and release of numerous mediators. Compromised glial activity coupled with a predisposing genetic background has been proposed to contribute to ASD pathogenesis (Zeidan-Chulia et al., 2014; Petrelli et al., 2016). Furthermore, studies on animal models are consistent with human observations demonstrating astrocyte abnormalities in ASD (Boldrini et al., 2018; Scuderi and Verkhratsky, 2020). For instance, some of the genes contributing to brain development and conferring susceptibility to ASD are highly expressed in astrocytes (Stogsdill et al., 2017; Sakers and Eroglu, 2019). Post-mortem brain samples of ASD subjects show abnormal levels of cytokines and chemokines together with signs of astrogliosis and microgliosis (Liao et al., 2020). Given the role of glia in regulating synaptic activity, a sustained presence of reactive glia could explain the region-specific altered connectivity seen in ASD patients, as well as their cognitive and behavioural traits (Just et al., 2007; Assaf et al., 2010; Supekar et al., 2013).

## Conclusions

Although COVID-19 and ASD differ in their aetiology and pathobiology, they share a single common feature: both are associated with the aberrant activation of the immune system and establishment of a pro-inflammatory environment. Growing evidence indicates the role of glial cells in both pathologies. The involvement of glia in the neurological consequences of COVID-19 has recently been documented, whereas the neuropathological potential of glia in ASD is established. No data are available yet on the consequences of foetal exposure to SARS-CoV-2 infection. However, coronaviruses, like SARS-CoV-2, have the potential to provoke adverse maternal or perinatal outcomes. Generally, maternal infection and fever during pregnancy double the risk of ASD in infants. Foetal exposure to infections is accompanied by an increased expression of markers of glia reactivity and proinflammatory mediators as well as an altered expression of genes involved in brain development. Therefore, at least hypothetically, SARS-CoV-2 infection may impair the baby's brain development by boosting cytokines circulation in the pregnant mother, potentially increasing the risk for ASD. The reactivity of neuroglia and in particular of astrocytes could mediate these adverse effects on the foetal brain.

The validity of this hypothesis is yet impossible to confirm because of the scarcity of data, and yet it is crucial to monitor babies born from mothers who suffered from COVID-19 during pregnancy, for the potential risk for ASD as well as other neurodevelopment pathologies.

## Author Contributions

MV, AV, and CS conceived and wrote the review manuscript. MV prepared the figure. All authors contributed to the design, writing, and revision of the paper.

## Funding

CS was supported by the Italian Ministry of University and Research (MUR) (PRIN prot. 2015KP7T2Y_002) and the SAPIENZA University of Rome (prot. RM11916B7A8D0225).

## Conflict of Interest

The authors declare that the research was conducted in the absence of any commercial or financial relationships that could be construed as a potential conflict of interest.

## Publisher's Note

All claims expressed in this article are solely those of the authors and do not necessarily represent those of their affiliated organizations, or those of the publisher, the editors and the reviewers. Any product that may be evaluated in this article, or claim that may be made by its manufacturer, is not guaranteed or endorsed by the publisher.
